# Dual Role of *PTPN22* but Not *NLRP3* Inflammasome Polymorphisms in Type 1 Diabetes and Celiac Disease in Children

**DOI:** 10.3389/fped.2019.00063

**Published:** 2019-03-12

**Authors:** Darja Smigoc Schweiger, Katja Goricar, Tinka Hovnik, Andrijana Mendez, Natasa Bratina, Jernej Brecelj, Blanka Vidan-Jeras, Tadej Battelino, Vita Dolzan

**Affiliations:** ^1^Department of Pediatric Endocrinology, Diabetes and Metabolic Diseases, University Children's Hospital, University Medical Centre Ljubljana, Ljubljana, Slovenia; ^2^Pharmacogenetics Laboratory, Faculty of Medicine, Institute of Biochemistry, University of Ljubljana, Ljubljana, Slovenia; ^3^Unit of Special Laboratory Diagnostics, University Children's Hospital, University Medical Centre Ljubljana, Ljubljana, Slovenia; ^4^Tissue Typing Centre, Blood Transfusion Center of Slovenia, Ljubljana, Slovenia; ^5^Faculty of Medicine, University of Ljubljana, Ljubljana, Slovenia; ^6^Department of Gastroenterology, Hepatology and Nutrition, University Children's Hospital, University Medical Centre Ljubljana, Ljubljana, Slovenia

**Keywords:** inflammasome, *NLRP3*, *CARD8*, *PTPN22*, type 1 diabetes, celiac disease

## Abstract

Genetic polymorphisms in genes coding for inflammasome components *nucleotide-binding oligomerization domain leucine rich repeat and pyrin domain-containing protein 3 (NLRP3)* and *caspase recruitment domain-containing protein 8 (CARD8)* have been associated with autoinflammatory and autoimmune diseases. On the other hand several studies suggested that NLRP3 inflammasome contributes to maintenance of gastrointestinal immune homeostasis and that activation of NLRP3 is regulated by protein tyrosine phosphatase non-receptor 22 (PTPN22). *PTPN22* polymorphism was implicated in the risk for various autoimmune diseases including type 1 diabetes (T1D) but not for celiac disease (CD). The aim of our study was to evaluate the role of inflammasome related polymorphisms in subjects with either T1D or CD as well as in subjects affected by both diseases. We examined *PTPN22* rs2476601 (p.Arg620Trp), *NLRP3* rs35829419 (p.Gln705Lys), and *CARD8* rs2043211 (p.Cys10Ter) in 66 subjects with coexisting T1D and CD, 65 subjects with T1D who did not develop CD, 67 subjects diagnosed only with CD and 127 healthy unrelated Slovenian individuals. All results were adjusted for clinical characteristic and human leukocyte antigen (HLA) risk. *PTPN22* rs2476601 allele was significantly more frequent among subjects with T1D (P_adj_ = 0.001) and less frequent in subjects with CD (P_adj_ = 0.039) when compared to controls. In patients with coexisting T1D and CD this variant was significantly less frequent compared to T1D group (P_adj_ = 0.010). Protective effect on CD development in individuals with T1D was observed only within the low risk HLA group. On the other hand, we found no association of *NLRP3* rs35829419 and *CARD8* rs2043211 with the development of T1D, CD or both diseases together. In conclusion *PTPN22* rs2476601polymorphism was significantly associated with the risk of developing T1D in Slovenian population, while no associations of proinflammatory *NLRP3* and *CARD8* polymorphisms with T1D and CD were observed. Interestingly, the same *PTPN22* variant protected from CD. We hypothesize that this effect may be mediated through the NLRP3 inflammasome activation.

## Introduction

Type 1 diabetes (T1D) is characterized by chronic autoimmune mediated destruction of pancreatic β-cells, leading to symptomatic partial, or in most cases, absolute insulin deficiency requiring lifelong administration of exogenous insulin (1). Celiac disease (CD) is a chronic immune-mediated disorder characterized by the presence of variable combination of clinical manifestations ranging from enteropathy to a systemic affliction of different organ systems and appearance of autoimmune antibodies elicited by ingestion of gluten and related prolamines (2). Both multifactorial diseases have a strong autoimmune and genetic component and often segregate together (3). Because of common association of T1D with CD, screening for CD is recommended in all children with T1D (1, 2). In individuals with T1D positive serum anti-tissue transglutaminase (tTG) antibodies may spontaneously normalize even on a gluten-containing diet (4). Therefore, duodenal biopsy is still required in asymptomatic individuals with T1D to confirm the diagnosis of CD (2). The majority of patients develop CD after the onset of T1D and the risk is significantly higher in children with T1D diagnosed before 5 years of age (5). In addition to CD autoimmune thyroid disease (ATD) is frequently seen in individuals with T1D (6). Having T1D and ATD has been reported to be a risk factor for the development of CD (5). On the other hand children affected by CD were at increased risk of subsequent hypothyroidism (7).

Shared susceptibility alleles in the Human leukocyte antigen (HLA) region encoding HLA-DQ2 and DQ8 molecules which account for much of the etiology of T1D and CD contribute to the coexistence of both diseases. The risk is further modified by the HLA genotype, which is the combination of HLA alleles inherited from both parents (8, 9). In our previous study, *DR3-DQ2/DR4-DQ8* and *DR3-DQ2/DR3-DQ2* genotypes were found to be highly predisposing for developing both diseases together (10). However, disease co-occurrence is greater that could be accounted for HLA-DR-DQ genotypes. In the prospective birth cohort study persistent islet autoantibodies were associated with increasing risk of subsequent development tTG antibodies, suggesting that T1D autoimmunity is a risk factor for subsequent CD (11). Cumulative genetic risk associated with both T1D and CD increases substantially when combined with the effect of genetic polymorphisms outside the HLA region (12). Among non-HLA factors shared pathophysiological mechanisms may involve many genes of the innate immune signaling, which is the first line of defense against invading microorganisms not only by directly eliminating the pathogen but also through further shaping of the adaptive immunity (13). It is hypothesized that the environment increasingly conducive to T1D development may potentiate autoimmunity by dysregulating innate immune processes in genetically susceptible individuals (14, 15). The inflammasomes are highly conserved pattern-recognition receptors expressed by cells of the innate immune system and some tissue cells. These cells recognize microbial pathogens as well as cellular stress and damage, leading to production of pro-inflammatory cytokines interleukin (IL)-1β and IL-18 and triggering a process of inflammation-related cell death (16). The best characterized is the NLRP3 inflammasome, the protein complex including nucleotide-binding oligomerization domain leucine rich repeat and pyrin domain-containing protein 3 (NLRP3) protein, adapter protein apoptosis-associated speck-like protein and procaspase-1. Interactions among these proteins tightly regulate inflammasome function in order to ensure immune activity only when appropriate (17). *NLRP3* rs35829419 (p.Gln705Lys) is a gain-of-function polymorphism associated with pro-inflammatory phenotype (18, 19). Caspase recruitment domain-containing protein 8 (CARD8) negatively regulates NLRP3 inflammasome through its binding with NLRP3 protein. *CARD8* rs2043211 (p.Cys10Ter) results in non-functional protein and leads to loss of CARD-8 inhibition of caspase-1 (20). Aberrant NLRP3 inflammasome activation has been linked to various immune disorders (21) and can also contribute to the onset and progression of metabolic diseases (22, 23). It was shown that NLRP3 played an important role in the immune pathogenesis of T1D development in NOD mice (24). It seems that NRLP3 also plays a regulatory and reparative role in the maintenance of immune tolerance and intestinal epithelial barrier integrity (25).

Tyrosine phosphorylation is an important regulatory mechanism that prevents aberrant inflammasome activation. A recent study has shown that protein tyrosine phosphatase non-receptor 22 (PTPN22) interacts with and dephosphorylates NLRP3 upon proinflammatory insults, allowing robust NLRP3 activation and IL-1β secretion (26). The *PTPN22* rs2476601 (p.Arg620Trp) polymorphism has been linked to several autoimmune diseases (27). The association between *PTPN22* rs2476601 and T1D was initially identified by Bottini et al. in two independent populations (28). Interestingly, the results of comprehensive meta-analysis showed no or weak association of *PTPN22* rs2476601 with CD (29, 30) and some autoimmune diseases primarily affecting the gastrointestinal system (31).

Previous studies have shown conflicting and heterogeneous findings with regard to the role of *NLRP3* and *PTPN22* polymorphisms in either T1D or CD onset. However, T1D and CD frequently occur in the same individual, pointing to shared mechanisms. The aim of our study was to investigate the role of *PTPN22* rs2476601 (p.Arg620Trp), *NLRP3* rs35829419 (p.Gln705Lys), and *CARD8* rs2043211 (p.Cys10Ter) in subjects with T1D who did not develop CD, in subjects with CD who did not develop T1D and in subjects who developed both diseases. Furthermore, the role of these polymorphisms was assessed while also taking into account the presence of high or low risk HLA genotype.

## Materials and Methods

### Study Design and Subjects

In total 325 individuals, who were previously HLA typed (10), were included in our study. Patients were stratified into three groups: 66 with coexisting T1D and CD (T1D+CD), 65 with T1D who did not develop CD after minimum of 10 years of follow up and 67 diagnosed only with CD. A group of 127 healthy unrelated Slovenian individuals served as a control group. In all subjects CD was diagnosed according to the relevant European Society for Pediatric Gastroenterology Hepatology and Nutrition (ESPGHAN) criteria (2, 32). In individuals with T1D serological screening for CD started at the onset of T1D and continued yearly. Study included 862 children from 0 to 17.99 years of age at diagnosis, referred to the University Children's Hospital Ljubljana, Slovenia at the onset of T1D, from 1995 to 2009. Screening for celiac disease was based on the detection of endomysial immunoglobulin A (IgA) autoantibodies (EMA) measured by indirect immunofluorescence until 2000. Afterwards tTG measured by enzyme linked immunosorbent assay were used for screening. Total serum IgA was used to exclude IgA deficiency. In IgA-deficient individuals IgG-specific anti-bodies (tTG and/or EMA IgG) were detected. Since individuals with T1D may have fluctuating levels of CD-specific antibodies, the definitive diagnosis of CD was established by an intestinal biopsy sample showing a Marsh score of 2 or higher in 69 participants. This study included 66 participants for whom DNA samples were available. Most of the subjects were diagnosed with CD after T1D diagnosis (designated as T1D first), while 22 (33.8%) had CD before T1D onset or were diagnosed with CD at the time of T1D diagnosis as the result of the screening (designated as CD first).

### *PTPN22, NLRP3*, and *CARD8* Genotyping

For the detection of *PTPN22* rs2476601 (c.1858T>C) polymorphism we used TaqMan SNP Genotyping assay (ID:C_16021387_20; Applied Biosystems, Foster City, CA, US). *NLRP3* rs35829419 (c.2113C>A, p.Gln705Lys) and *CARD8* rs2043211 (c.304A>T; p.Phe102Ile, p.Cys10Ter) were genotyped using a fluorescence-based competitive allele-specific (KASPar) assay according to the manufacturer's instructions (KBiosciences, Herts, UK). Genotyping was repeated in 20% of samples to check for genotyping reliability.

### *HLA DRB1, DQB1*, and *DQA1* Typing

The *DRB1, DQB1*, and *DQA1* genotyping was performed as previously described (10). We performed PCR-sequence-specific primer (PCR-SSP) typing (Olerup SSP, Stockholm, Sweden), PCR-reverse sequence-specific oligonucleotide probe (PCR-rSSO) typing assay (LabType SSO, One Lambda, Kanoga Park, CA, USA) with Luminex technology (LABSCAN TM 100, Luminex corporation, Austin, TX, USA) and PCR-sequencing based typing (PCR-SBT) (Conexio's SBT Resolver™, Conexio-Genomics, Fremantle, Western Australia).

### Statistical Analysis

HLA alleles were deduced into three loci haplotypes (*DRB1-DQA1-DQB1*) as described earlier (10). DRB1^*^03:01-DQA1^*^05:01-DQB1^*^02:01 haplotype was abbreviated as DR3-DQ2, DRB1^*^07:01–DQA1^*^02:01-DQB1^*^02:02 as DR7-DQ2 and DRB1^*^11/12–DQA1^*^05:05–DQB1^*^03:01 as DR5-DQ7. DRB1^*^04:01-DQA1^*^03:01-DQB1^*^03:02, DRB1^*^04:02-DQA1^*^03:01-DQB1^*^03:02, DRB1^*^04:04-DQA1^*^ 03:01-DQB1^*^03:02, DRB1^*^04:05-DQA1^*^03:01-DQB1^*^03:02, and DRB1^*^04:08-DQA1^*^03:01-DQB1^*^03:04 haplotypes were considered as DR4-DQ8. X was defined as non-DR3-DQ2 and non-DR4-DQ8 haplotype. *HLA-DRB1-DQA1-DQB1/DRB1-DQA1-DQB1* genotypes (combination of two *DRB1-DQA1-DQB1* haplotypes) were grouped into two HLA-risk categories encoded as low-risk (LR) and high-risk (HR) categorical variables by means of the known risk associated with T1D and CD in the Slovenian population. The heterozygous genotype DR3-DQ2/DR4-DQ8 and genotype DR4-DQ8/ DR4-DQ8 were defined as HR for T1D (8, 33, 34). Since the HLA influence on CD susceptibility showed a dose effect, DR3-DQ2/ DR3-DQ2 and DR3-DQ2/ DR7-DQ2, which are DQB1^*^02 homozygous combinations were classified as high risk for CD (10). DR3-DQ2/X *and* DR4-DQ8/X single dose combinations, DRB1^*^07-DQA1^*^02:01-DQB1^*^02:02/DRB1^*^11/12-DQA1^*^05:05-DQB1^*^03:01 haplotype combination with DQ2 alleles carried in trans as well as non-DQ2 and non-DQ8 (X/X) haplotype combinations were considered as low risk.

For the description of continuous and categorical variables, median with interquartile range and frequencies were used, respectively. Standard chi square test was used to compare distribution of categorical clinical characteristics between different patient groups and to assess deviation from Hardy-Weinberg equilibrium. Nonparametric Mann-Whitney and Kruskal-Wallis tests were used to compare continuous clinical characteristics between different patient groups. Logistic regression was used for comparison of genotype frequencies, calculating odds ratio (OR) and 95% confidence interval (CI), as well as for the analysis of interaction between polymorphisms. Two-tailed Fisher's exact test was used for comparisons of the frequencies of HLA deduced genotypes. The level of statistical significance was set at 0.05. All statistical analyses were performed using IBM SPSS Statistics 19.0 (IBM Corporation, Armonk, NY, USA).

## Results

### Clinical Characteristics of Subjects With T1D, CD, and T1D+CD

Clinical characteristics of subjects with T1D, CD, or both including HLA-risk categories are shown in Table 1. Subjects with CD were the youngest at the time of the first diagnosis, but the difference did not reach statistical significance when all three groups were compared (*P* = 0.072, Supplementary Figure 1). Antithyroid antibodies were observed in 18 individuals with T1D (27.7%) and in 16 individuals (24.2%) with coexisting T1D and CD with no significant difference between the two groups. Three individuals (4.5%) were diagnosed with ATD in the CD only group (Table 1).

**Table 1:** Characteristics of subject with T1D, CD, and T1D+CD.

**Characteristic**	**T1D** ***N* = 65**	**CD** ***N* = 67**	**T1D + CD** ***N* = 66**
**GENDER**			
Male *N* (%)	32 (49.2)	23 (34.3)	32 (48.5)
Female *N* (%)	33 (50.8)	44 (65.7)	34 (51.5)
**AGE AT T1D DIAGNOSIS**			
Median (25–75%)	7.8 (3.6–11.4)		8.6 (4.0–11.8)
**AGE AT CD DIAGNOSIS**			
Median (25–75%)		4.8 (2.3–9.5)	10.0 (5.0–14.9)
**CD DIAGNOSIS BEFORE/AT T1D DIAGNOSIS**			
^a^*N* (%)			22 (33.8)
ATD (%)	18 (27.7)	3 (4.5)	16 (24.2)
**HLA GENOTYPE**			
LR *N* (%)	39 (60.0)	43 (64.2)	28 (42.4)
HR *N* (%)	26 (40.0)	24 (35.8)	38 (57.6)

*T1D, type 1 diabetes; CD, celiac disease; T1D+CD, coexisting T1D and CD; ATD, autoimmune thyroid disease; HR, high risk HLA genotype for T1D or CD; LR, low risk HLA genotype for T1D or CD*.

a *Data on first diagnosis is missing for 1 subject with coexisting T1D and CD*.

### *HLA-DRB1-DQA1-DQB1* Deduced Genotype Frequencies in Individuals With Coexisting T1D and CD

The distribution of HLA genotypes differed significantly among the groups (Supplementary Table 1). Two high-risk genotypes DR3-DQ2/DR4-DQ8 (*p* < 0.001) and DR3-DQ2/DR3-DQ2 (*p* < 0.001) were associated with an increased risk for developing the combination of both diseases. On the other hand low risk genotypes had neutral effect on co-occurrence. When individuals with T1D who later developed CD were compared to those with T1D alone, genotype DR3-DQ2/DR3-DQ2 (OR = 7.10, *p* = 0.002) conferred an increased risk for CD and DR4-DQ8/X (OR = 0.19, *p* = 0.001) was negatively associated. Comparisons of individuals in whom CD diagnosis proceeded T1D with CD only group revealed that DR3-DQ2/DR4-DQ8 genotype was associated with an increased risk for developing T1D (OR = 9.96, *p* = 0.002).

### *PTPN22, NLRP3*, and *CARD8* Genotype Frequencies Distribution

Genotype frequencies were in agreement with HWE in the control group (*P* = 0.413 for *PTPN22* rs2476601, *P* = 0.461 for *NLRP3* rs35829419, and *P* = 0.616 for *CARD8* rs2043211) as well as in the entire study group (*P* = 0.823 for *PTPN22* rs2476601, *P* = 0.138 for *CARD8* rs2043211, and *P* = 0.607 for *NLRP3* rs35829419). Minor allele frequencies were 14.2% for *PTPN22* rs35829419, 38.2% for *CARD8* rs2043211, and 6.6% for *NLRP3* rs2476601 (Table 2).

**Table 2:** Genotype frequencies of *CARD8, NLRP3*, and *PTPN22* polymorphisms.

**Polymorphism**	**Genotype**	**All individuals *N* (%)**	**Controls** ***N* (%)**	**T1D** ***N* (%)**	**CD** ***N* (%)**	**T1D + CD** ***N* (%)**	**T1D first^a^** ***N* (%)**	**CD first^a^** ***N* (%)**
***CARD8*** **rs2043211**	AA	118 (36.3)	51 (40.2)	26 (40.0)	20 (29.9)	21 (31.8)	15 (34.9)	6 (27.3)
	AT	166 (51.1)	61 (48.0)	31 (47.7)	36 (53.7)	38 (57.6)	25 (58.1)	13 (59.1)
	TT	41 (12.6)	15 (11.8)	8 (12.3)	11 (16.4)	7 (10.6)	3 (7.0)	3 (13.6)
***NLRP3*** **rs35829419**^**b**^	CC	283 (87.3)	111 (88.1)	59 (90.8)	58 (86.6)	55 (83.3)	36 (83.7)	18 (81.8)
	CA	39 (12.0)	14 (11.1)	5 (7.7)	9 (13.4)	11 (16.7)	7 (16.3)	4 (18.2)
	AA	2 (0.6)	1 (0.8)	1 (1.5)	0 (0.0)	0 (0.0)	0 (0.0)	0 (0.0)
***PTPN22*** **rs2476601**	CC	240 (73.8)	96 (75.6)	35 (53.8)	60 (89.6)	49 (74.2)	30 (69.8)	18 (81.8)
	CT	78 (24.0)	30 (23.6)	29 (44.6)	6 (9.0)	13 (19.7)	12 (27.9)	1 (4.5)
	TT	7 (2.2)	1 (0.8)	1 (1.5)	1 (1.5)	4 (6.1)	1 (2.3)	3 (13.6)

*T1D, type 1 diabetes; CD, celiac disease; T1D+CD, coexisting T1D and CD; T1D first, patients who first developed T1D and later CD; CD first, patients who first developed CD and later T1D*.

a *Data on first diagnosis is missing for 1 subject with coexisting T1D and CD*.

b *Data is missing for 1 control*.

### Association Between *PTPN22, NLRP3*, and *CARD8* Genotype Frequencies and Risk of T1D, CD, and T1D+CD

Proportion of carriers of at least one polymorphic *PTPN22* rs2476601allele was significantly higher among subjects withT1D (OR = 2.65, 95% CI = 1.41–5.00, *P* = 0.003) and the difference remained statistically significant after adjustment for gender (OR = 3.02, 95% CI = 1.54–5.93, *P* = 0.001). On the other hand, polymorphic *PTPN22* rs2476601allele was significantly less common in subjects with CD when compared to controls (OR = 0.36, 95% CI = 0.15–0.87, *P* = 0.024), the difference remained significant after adjustment for gender (OR = 0.39, 95% CI = 0.16–0.96, *P* = 0.039). There were no significant differences in genotype frequencies for *NLRP3* rs35829419 and *CARD8* rs2043211 between cases and controls, not even after adjustment for clinical parameters (Table 3).

**Table 3:** Association between *CARD8, NLRP3, and PTPN22* genotype frequencies and risk of T1D, CD and T1D+CD.

		**T1D vs. controls**		**CD vs. controls**		**T1D+CD vs. controls**	
		**OR** **(95% CI)**	***P***^a^	**OR** **(95% CI)**	***P*^a^**	**OR** **(95% CI)**	***P*^a^**
*CARD8* rs2043211	AT	0.96 (0.49–1.87)	0.900	1.45 (0.75–2.82)	0.274	1.34 (0.68–2.64)	0.397
	TT	1.11 (0.40–3.08)	0.842	1.89 (0.73–4.84)	0.188	1.18 (0.41–3.43)	0.761
	AT+TT	0.99 (0.52–1.87)	0.967	1.54 (0.81–2.91)	0.187	1.31 (0.62–2.52)	0.417
*NLRP3* rs35829419	CA+AA	0.76 (0.27–2.15)	0.609	1.20 (0.49–2.93)	0.692	1.53 (0.64–3.68)	0.343
*PTPN22* rs2476601	CT+TT	3.02 (1.54–5.93)	0.001	0.39 (0.16–0.96)	0.039	1.25 (0.61–2.56)	0.543

*T1D, type 1 diabetes; CD, celiac disease; T1D+CD, coexisting T1D and CD*.

a *Adjusted for gender*.

### Comparison of *PTPN22, NLRP3*, and *CARD8* Genotype Frequencies of Subjects With T1D, CD, and T1D+CD

Proportion of carriers of at least one polymorphic *PTPN22* rs2476601allele in subjects with coexisting T1D and CD was significantly lower compared to T1D (OR = 0.41, 95% CI = 0.19–0.85, *P* = 0.016) and the difference remained significant after adjustment for age at diagnosis, gender and HLA genotype (OR = 0.36, 95% CI = 0.17–0.78, *P* = 0.010). Proportion of carriers of at least one polymorphic *PTPN22* rs2476601 allele in subjects with coexisting T1D and CD was significantly higher compared to CD (OR = 2.97, 95% CI = 1.14–7.75, *P* = 0.026), however the difference was no longer significant after adjustment for age at diagnosis, gender and HLA genotype (OR = 2.42, 95% CI = 0.82–7.12, *P* = 0.109). *NLRP3* rs35829419 and *CARD8* rs2043211 genotype frequencies did not differ significantly among subjects with T1D, CD, or coexisting diseases (Table 4). No significant differences in genotype frequencies were observed when subjects with coexisting T1D and CD were stratified according to the order of the disease onset (T1D first and CD first groups) and compared to patients with T1D only and CD only as well as when compared between themselves (Table 4).

**Table 4:** Comparison of *CARD8, NLRP3*, and *PTPN22* genotype frequencies of subjects with T1D, CD, and T1D+CD.

		**T1D+CD vs. T1D**		**T1D+CD vs. CD**		**T1D first vs. T1D**		**CD first vs. CD**		**CD first vs. T1D first**	
		**OR** **(95% CI)**	***P*^a^**	**OR** **(95% CI)**	***P*^b^**	**OR** **(95% CI)**	***P*^a^**	**OR** **(95% CI)**	***P*^b^**	**OR** **(95% CI)**	***P*^c^**
*CARD8* rs2043211	AT	1.39 (0.65–2.98)	0.402	0.89 (0.38–2.09)	0.786	1.24 (0.53–2.88)	0.622	1.32 (0.41–4.28)	0.641	1.68 (0.49–5.76)	0.406
	TT	0.97 (0.30–3.17)	0.954	0.58 (0.16–2.10)	0.411	0.60 (0.14–2.68)	0.504	0.99 (0.19–5.10)	0.994	2.45 (0.36–16.81)	0.362
	AT+TT	1.30 (0.62–2.71)	0.486	0.82 (0.36–1.87)	0.636	1.11 (0.49–2.51)	0.808	1.25 (0.40–3.88)	0.705	1.80 (0.55–5.90)	0.334
*NLRP3* rs35829419	CA+AA	2.01 (0.68–5.95)	0.210	1.44 (0.50–4.09)	0.500	1.89 (0.57–6.25)	0.299	1.22 (0.31–4.79)	0.778	1.08 (0.27–4.34)	0.917
*PTPN22* rs2476601	CT+TT	0.36 (0.17–0.78)	0.010	2.42 (0.82–7.12)	0.109	0.47 (0.20–1.11)	0.085	1.88 (0.48–7.41)	0.367	0.25 (0.11–1.77)	0.251

*HR, high risk HLA genotype; LR, low risk HLA genotype; T1D, type 1 diabetes; CD, celiac disease; T1D+CD, coexisting T1D and CD; T1D first, patients who first developed T1D and later CD; CD first, patients who first developed CD and later T1D*.

a *Adjusted for gender, age at T1D diagnosis, HLA HR vs. LR*.

b *Adjusted for gender, age at CD diagnosis, HLA HR vs. LR*.

c *Adjusted for gender, age at first diagnosis, HLA HR vs. LR*.

### Multiplicative Interaction

When comparing subjects that were first diagnosed with T1D and then developed CD (T1D first) to patients with only T1D, we observed a multiplicative interaction between *PTPN22* and HLA (OR = 7.19, 95% CI = 1.03–50.29, *P* = 0.047) (Table 5). Subjects with LR HLA and *PTPN22* CC genotypes were the reference category. Carriers of at least one polymorphic *PTPN22* allele were significantly less frequent among subjects withT1D first compared to T1D only within HLA LR group (*P* = 0.015), but not in the HLA HR group. Among carriers of at least one polymorphic *PTPN22* allele, T1D first were more likely HLA HR than T1D (*P* = 0.014). A similar trend was observed when comparing group of subjects with both T1D and CD (T1D first + CD first) to subjects with only T1D or CD, but it did not reach statistical significance. No multiplicative interaction was observed between *NLRP3* rs35829419 and *CARD8* rs2043211 (Supplementary Table 2).

**Table 5:** Interaction between *PTPN22* rs2476601 and HLA (comparing T1D first to T1D).

	**HLA**								**HLA within one** ***PTPN22*** **category**	
	**LR**				**HR**					
***PTPN22***	**T1D first** **(*N*)**	**T1D** **(*N*)**	**OR** **(95% CI)**	***P***	**T1D first** **(*N*)**	**T1D** **(*N*)**	**OR** **(95% CI)**	***P***	**OR** **(95% CI)**	***P***
CC	17	21	1	Ref.	13	14	1.15 (0.43–3.09)	0.786	1.15 (0.43–3.09)	0.786
CT+TT	2	18	0.14 (0.03–0.68)	0.015	11	12	1.13 (0.40–3.20)	0.815	8.25 (1.55–44.02)	0.014
*PTPN* within one HLA category			0.14 (0.03–0.68)	0.015			0.99 (0.32–3.00)	0.982		

*T1D, type 1 diabetes; CD, celiac disease; T1D+CD, coexisting T1D and CD; T1D first, patients who first developed T1D and later CD; HR, high risk HLA genotype; LR, low risk HLA genotype*.

## Discussion

The present study investigated the role of *PTPN22* and inflammasome polymorphisms in the risk for development of T1D and CD as separate or concurrent diseases. Our results suggest a dual role of the *PTPN22* polymorphism in the development of autoimmune diseases: while rs2476601 polymorphism was associated with increased risk for T1D, it was protective against development of CD. On the other hand, *NLRP3* and *CARD8* polymorphisms were not associated with the risk of either T1D or CD.

Previous studies reported associations of *PTPN22* and *NLRP3* polymorphisms with several autoimmune diseases suggesting their general role in the etiology of autoimmunity, however these risk alleles are not shared among all autoimmune disease (31, 35). NOD mice models showed an important role of aberrant NLRP3 activation in the immune pathogenesis of T1D (24). In preclinical studies NLRP3 deficient animals have been shown to have defective epithelial barrier function and increased intestinal inflammation (36). In human studies of CD, peripheral blood mononuclear cells and monocytes responded to gliadin fraction by a robust secretion of IL-1β and IL-1α and a slightly elevated production of IL-18 (37). In contrast with these functional studies, our results did not confirm the role of functional *NLRP3* rs35829419 and *CARD8* rs2043211 polymorphisms that promote NLRP3 inflammasome activation in the risk of either T1D or CD. Conflicting results were also reported in the literature, as the minor *NLRP3* rs35829419 A allele was reported to be associated with CD in Brazilian population (38), however in Italian patients this allele appeared to have a protective role against the development of CD (39).

Our finding that *PTPN22* polymorphism has a dual role in the development of autoimmune diseases is of particular interest as recent studies showed that NLRP3 activation is negatively regulated by tyrosine phosphorylation (40). The presence of autoimmunity associated PTPN22 variant in mice (PTPN22-619W) resulted in dephosphorylation and activation of NLRP3, while loss of PTPN22 resulted in decreased NLRP3 mediated IL-1β secretion. PTPN22 affected NLRP3 activation only in the lamina propria, resulting in only minor changes of IL-18 activation, which is mainly produced by intestinal epithelial cells. Furthermore, loss of PTPN22 resulted in aggravated intestinal inflammation (26). Of note *PTPN22* c.1858C>T variant leading to p.Arg620Trp substitution (rs2476601) showed association pattern with target tissue specificity being less involved in pathogenesis of autoimmune diseases triggered by environmental factors primarily acting on target tissues such as gut mucosa (31). Supporting the notion of negligible effect of *PTPN22* rs2476601 on CD development, no statistically significant association of this polymorphism with CD was found in a north European (41, 42) and Spanish population (29). Nevertheless, we observed an apparent protective effect of *PTPN22* rs2476601 polymorphism on CD development, suggesting a role of PTPN22 in facilitating inflammasome activation in intestinal lamina propria.

Previous studies have consistently associated *PTPN22* rs2476601 with the T1D onset in several populations (43). A definite effect of this polymorphism on the appearance of autoantibodies and progression to clinical T1D was observed in the DIPP study cohort, where the carriers of the *PTPN22* rs2476601 polymorphism showed enhanced appearance of T1D associated autoantibodies when exposed to cow milk in early infancy (44). Consistent with these reports we observed significantly higher frequency of carriers of at least one rs2476601 T allele among subjects withT1D without associated CD as compared to the control group supporting a role of this polymorphism in disease susceptibility in the Slovenian population. Given a role of PTPN22 in pathogenesis of the T1D and lack of association to CD we also investigated the role of *PTPN22* in T1D with accompanying CD. However, unlike to the individuals with isolated T1D, *PTPN22* rs2476601 was not associated with increased risk for T1D with accompanying CD. These results suggest that *PTPN22* rs2476601 may not influence T1D pathophysiological mechanisms in all cases, while previous reports on the association of this variant and APSIII onset suggest common pathogenic pathways (45). The discrepancies observed among studies may in part be explained by considerable differences of *PTPN22* rs2476601 frequencies in populations of European origin (31). Minor allele frequency for *PTPN22* rs2476601 in our population was 14.2%, resembling more closely those from northern European populations, which are among the highest in Europe (46).

Since HLA remains, by far, the strongest predictor of T1D and CD risk all results in our study were adjusted for HLA risk and only odds ratios adjusted for given HLA risk category were reported. Previous studies have shown that the strength of association between *PTPN22* rs2476601 and T1D is stronger in low-risk HLA subjects, compared with that in subjects caring high-risk HLA genotypes (47). In our study, the interaction effect of HLA-risk category on the association of *PTPN22* rs2476601 was observed when individuals who had first T1D and then developed CD (T1D first group) were compared to individuals with T1D who did not develop CD. Protective effect on CD development was observed within the low risk HLA category but no significant difference was observed between groups within the HLA high risk category. More than half of patients who had coexisting T1D and CD carried the high risk HLA-DR3-DQ2/DR3-DQ2 or HLA-DR3-DQ2/DR4-DQ8 genotype. On the other hand, DR4-DQ8/X was protective for developing CD in individuals with T1D. If the results could be confirmed on a larger number of patients of different ethnic origin, assessing the presence of HLA-DQ2 and DQ8 genotypes in individuals negative for celiac disease-specific antibodies at the time of T1D diagnosis could be considered as an algorithm tool to optimize further screening frequency. Yearly screening for CD may be considered in T1D individuals with high risk HLA-DR3-DQ2/DR3-DQ2 or HLA-DR3-DQ2/DR4-DQ8 genotypes. On the other hand, individuals carrying DR4-DQ8/X genotype could be further genotyped for PTPN22 rs2476601 (c.1858T>C) polymorphism and if positive, less frequent screening for CD may be suggested.

The small sample size was a limitation of our study. The possibility of insufficient power to detect associations of genetic variants with small effect on disease susceptibility must be considered. Our results suggest opposite effects of *PTPN22* variant on T1D and CD susceptibility and provide interesting information on incidence of this variant in subjects diagnosed with both autoimmune diseases together. To better understand the complex effect of genetic factors and their regulatory role in the pathogenesis of autoimmune diseases larger studies and functional studies are warranted. Due to the low prevalence of subjects with concurrently diagnosed T1D and CD the main advantage of our study was the use of longitudinal data (48) on the entire pediatric T1D population form the prospective Slovene childhood-onset T1D register (49) from 1995 to 2009, which allowed a longer follow up, up to 10 years for T1D only group.

In conclusion this study showed that *PTPN22* rs2476601 polymorphism was significantly associated with the risk of developing T1D in Slovenian population, and of interest, the same variant protected from CD. We hypothesize that the underlying mechanism for this controversial effect could be explained considering that NLRP3 activation is involved in maintaining intestinal homeostasis. However, no association of proinflammatory *NLRP3* and *CARD8* polymorphisms with CD were observed. Interestingly, *PTPN22* rs2476601 did not influence T1D susceptibility in individuals with coexisting CD, suggesting heterogeneity in the disease-predisposing effect of *PTPN22* rs2476601 in T1D with regard to the presence of CD. Although individuals with coexisting T1D and CD represent only a small percentage of all T1D cases, acknowledging the diversity of genetic background may help to improve immune intervention strategies aimed at curing T1D.

## Statement of Prior Presentation

Part of this study was presented as a poster with a title of “Polymorphism c1858t in the protein tyrosine phosphatase non-receptor type 22 in co-occurrence of type 1 diabetes and celiac disease” (Abstract Code: A-910-0012-00527) at the 43^rd^ Annual Conference of the International Society for Pediatric and Adolescent Diabetes in Innsbruck, Austria, October 18–21, 2017.

## Ethics Statement

The study protocol was approved by the Republic of Slovenia National Medical Ethics Committee (No. 98/09/12) and written informed consent was obtained from all participating individuals and/or their parents, when appropriate, prior to the enrolment.

## Author Contributions

DS collected clinical data, interpreted data, and drafted the manuscript. KG carried out the *NLRP3* and *CARD* molecular genetic studies, statistical analysis, and edited the manuscript. TH carried out the *PTPN22* molecular genetic studies. AM and BV-J performed analysis of the HLA genetic studies. NB and JB were responsible for the clinical data and biological sample collection used in this study. VD and TB contributed to the study design and conception and edited the manuscript. All the authors collaborated in revising the manuscript and accepted the final version of the manuscript.

### Conflict of Interest Statement

The authors declare that the research was conducted in the absence of any commercial or financial relationships that could be construed as a potential conflict of interest.

## Abbreviations

T1D: type 1 diabetes
CD: celiac disease
T1D+CD: coexisting T1D and CD
HLA: human leukocyte antigen
*NLRP3*: *nucleotide-binding oligomerization domain leucine rich repeat and pyrin domain-containing protein 3*
*CARD8*: *caspase recruitment domain-containing protein 8*
*PTPN22*: *protein tyrosine phosphatase non-receptor 22*
LR: low-risk
HR: high-risk.
