# Supplementary material for: Dual Role of PTPN22 but Not NLRP3 Inflammasome Polymorphisms in Type 1 Diabetes and Celiac Disease in Children
Source: Front Pediatr. 2019 Mar 12;7:63. doi: 10.3389/fped.2019.00063 (PMC6422865; doi:10.3389/fped.2019.00063)
Supplement: Supplementary file 1 [file Data_Sheet_1.pdf]

Supplementary Material

Dual role of *PTPN22* but not *NLRP3* inflammasome polymorphisms in type 1 diabetes and celiac disease in children

Darja Smigoc Schweiger[1], Katja Goricar[2], Tinka Hovnik[3], Andrijana Mendez[4], Natasa Bratina[1,6], Jernej Brecelj[5,6], Blanka Vidan-Jeras[4], Tadej Battelino[1,6], Vita Dolzan[2] *

[1] Department of Pediatric Endocrinology, Diabetes and Metabolic Diseases, University Children's Hospital, University Medical Centre Ljubljana, Ljubljana, Slovenia

[2] Pharmacogenetics Laboratory, Institute of Biochemistry, Faculty of Medicine, University of Ljubljana, Ljubljana, Slovenia

[3] Unit of Special Laboratory Diagnostics, University Children’s Hospital, University Medical Centre Ljubljana, Ljubljana, Slovenia

[4] Tissue Typing Centre, Blood Transfusion Center of Slovenia, Ljubljana, Slovenia

[5] Department of Gastroenterology, Hepatology and Nutrition, University Children's Hospital, University Medical Centre Ljubljana, Ljubljana, Slovenia

[6] Faculty of Medicine, University of Ljubljana, Ljubljana, Slovenia

*** Correspondence:**
Vita Dolzan: vita.dolzan@mf.uni-lj.si

Supplementary Figure 1

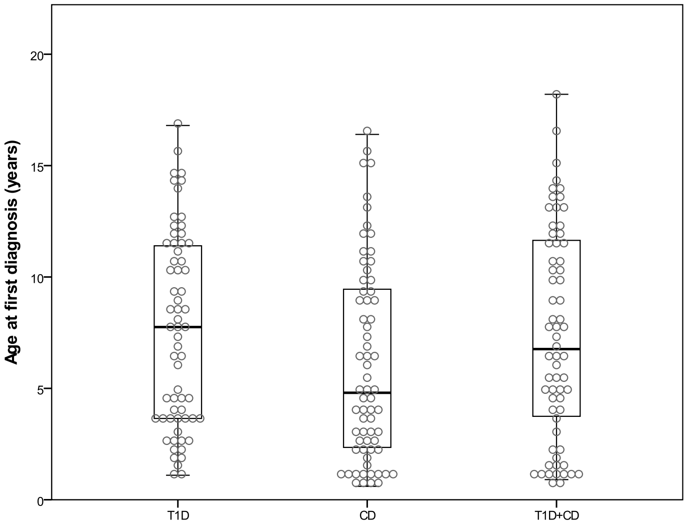

Supplementary Figure 1. Age at first diagnosis for different patient groups. Abbreviations: T1D, type 1 diabetes; CD, celiac disease; T1D+CD, coexisting T1D and CD.

Supplementary Table 1. Comparison of *HLA-DRB1-DQA1-DQB1* genotype frequencies.

	Controls	T1D	CD	T1D first[b]	CD first	T1D+CD vs controls		T1D first vs T1D		CD first vs CD	
	N (%)	N (%)	N (%)	N (%)	N (%)	OR (95% CI)	P	OR (95% CI)	P	OR (95% CI)	P
DR3-DQ2/DR3-DQ2	1 (0.8)	3 (4.6)	9 (13.4)	11 (25.6)	6 (27.3)	47.25 (6.14-363.72)	<0.001	7.10 (1.85-27.29)	0.002	2.42 (0.75-7.80)	0.187
DR3-DQ2/DR4-DQ8	0 (0.0)	17 (26.2)	3 (4.5)	10 (23.3)	7 (31.8)	/	<0.001	0.86 (0.35-2.10)	0.822	9.96 (2.30-43.07)	0.002
DR3-DQ2/DR7-DQ2	2 (1.6)	0 (0.0)	11 (16.4)	3 (7.0)	0 (0.0)	2.98 (0.49-18.27)	0.340	/	0.060	/	0.059
DR3-DQ2/X[a]	24 (18.9)	10 (15.4)	33 (49.3)	14 (32.6)	6 (27.3)	1.87 (0.94-3.71)	0.103	2.66 (1.05-6.72)	0.057	0.39 (0.14-1.11)	0.087
DR4-DQ8/DR4-DQ8	0 (0.0)	6 (9.2)	1 (1.5)	0 (0.0)	0 (0.0)	/	/[c]	/	0.079	/	1.000
DR4-DQ8/X	15 (11.8)	27 (41.5)	3 (4.5)	5 (11.6)	2 (9.1)	0.88 (0.34-2.29)	1.000	0.19 (0.06-0.53)	0.001	2.13 (0.33-13.68)	0.59
DR7-DQ2/DR5-DQ7	3 (2.4)	1 (1.5)	4 (6.0)	0 (0.0)	1 (4.5)	0.64 (0.07-6.24)	1.000	/	1.000	0.75 (0.08-7.09)	1.000
X/X	82 (64.6)	1 (1.5)	3 (4.5)	0 (0.0)	0 (0.0)						

T1D, type 1 diabetes; CD, celiac disease; T1D+CD, coexisting T1D and CD; T1D first, patients who first developed T1D and later CD; CD first, patients who first developed CD and later T1D.

[a] *X includes non-DR3-DQ2 and non-DR4-DQ8 haplotypes.*

[b] *Data on first diagnosis is missing for 1 subject with coexisting T1D and CD.*

[c] *Genotype not present in either of the groups.*

Supplementary Table 2. Interaction between *CARD8* rs2043211 and *NLRP3* rs35829419 (comparing all cases to controls).

	NLRP3								***NLRP3* within one *CARD8* category**	
	**CC**				**CA+AA**					
CARD8	**cases (N)**	**controls (N)**	**OR (95% CI)**	**P**	**cases (N)**	**controls (N)**	**OR (95% CI)**	**P**	**OR (95% CI)**	**P**
**AA**	55	47	1	Ref.	12	4	2.56 (0.78-8.48)	0.123	2.56 (0.78-8.48)	0.123
**AT+TT**	117	64	1.56 (0.95-2.56)	0.077	14	11	1.09 (0.45-2.62)	0.852	0.70 (0.30-1.62)	0.402
***CARD8* within one *NLRP3* category**			1.56 (0.95-2.56)	0.077			0.42 (0.11-1.69)	0.223		

T1D, type 1 diabetes; CD, celiac disease; T1D+CD, coexisting T1D and CD.
